# Validation of the Rehabilitation Activity Time Score in Mechanically Ventilated Intensive Care Unit Patients

**DOI:** 10.7759/cureus.102789

**Published:** 2026-02-01

**Authors:** Shinichi Watanabe, Mika Ono, Keisuke Suzuki, Takayasu Koike, Kenji Tsujimoto, Yasunari Morita

**Affiliations:** 1 Department of Rehabilitation Medicine, Gifu University of Health Science, Gifu, JPN; 2 Department of Nursing, Kanazawa Medical Center, Kanazawa, JPN; 3 Department of Physical Therapy, Gifu University of Health science, Gifu, JPN; 4 Department of Rehabilitation Medicine, Ichinomiyanishi Hospital, Aichi, JPN; 5 Department of Emergency Medicine, Nagoya Medical Center, Nagoya, JPN

**Keywords:** accelerometry, convergent validity, early mobilization, feasibility, intensive care unit, rehabilitation dose

## Abstract

Introduction

Quantifying the mobilization dose in the intensive care unit (ICU) remains a major challenge. Although the Mobilization Quantification Score (MQS) is a validated and widely used index, its calculation requires separate quantification of the time spent at multiple mobilization levels, making it complex and less feasible for routine clinical use. To address this limitation, we developed the Rehabilitation Activity Time score (RATs), which is a simple, time-based bedside tool designed to efficiently quantify the rehabilitation dose. This pilot study primarily aimed to evaluate the feasibility and convergent validity of RATs in mechanically ventilated ICU patients, and secondarily to explore their association with clinical outcomes.

Methods

This prospective single-center observational pilot study included adult ICU patients expected to require invasive mechanical ventilation for ≥48 hours and to receive rehabilitation as part of usual care. Daily RATs and MQS were recorded by physical therapists, and physical activity was continuously measured using a wrist-worn triaxial accelerometer (ActiGraph wGT3X-BT; Ametis, Pensacola, Florida). Feasibility outcomes included data completion rates, valid accelerometer wear days, and adverse events. Convergent validity among RATs, MQS, and accelerometer-derived activity metrics was assessed using Spearman's rank correlation. Exploratory analyses examined associations between activity measures and clinical outcomes.

Results

Twelve patients were screened, and 10 were enrolled (median age 68, IQR 61-81). Across 59 ICU patient-days, the RATs and MQS were recorded on 56 (95%) and 53 (90%) days, respectively, whereas accelerometry provided 55 valid days (83%). No device-related adverse events were observed. RATs was strongly correlated with the MQS (r=0.94) and accelerometer activity counts (r=0.76). Exploratory analyses indicated that higher mean RATs were associated with a higher discharge Barthel Index (r=0.56) and earlier ventilator liberation (median four vs. six days; Cliff delta =0.47).

Conclusion

The RATs demonstrated high feasibility and strong validity, reflecting objective activity levels while requiring minimal bedside effort. As a simple and reliable measure, the RATs may enable the standardized quantification of rehabilitation doses in the ICU and inform future dose-response research.

## Introduction

Early mobilization and rehabilitation in the intensive care unit (ICU) have been associated with improved functional outcomes, reduced delirium, and shortened the length of stay of critically ill patients, including those receiving mechanical ventilation [1-3]. As these interventions become standard care, accurately quantifying the "dose" and "intensity" of rehabilitation has become increasingly important. The Mobilization Quantification Score (MQS), which combines activity level and duration, is associated with clinical outcomes [4, 5]. However, its calculation is time-consuming and requires detailed documentation, limiting its feasibility in daily clinical practice. Therefore, a simpler and more clinically implementable tool with a comparable validity is required.

ICU patients frequently develop muscle weakness and ICU-acquired weakness, which contribute to long-term functional disability and reduced quality of life [6]. Adequate and timely physical activity is essential for preventing deconditioning and promoting recovery [2,3]. Although early physical and occupational therapies improve physical function and independence [3], qualitative and quantitative assessment of activity remains challenging [7]. To address this issue, we developed a Rehabilitation Activity Time score (RATs), a time-based bedside measure that allows immediate recording of activity levels and durations. RATs has demonstrated high inter-rater reliability and a very strong correlation with the MQS, suggesting comparable validity with greater simplicity and clinical applicability [8].

Wearable accelerometers (e.g., ActiGraph) provide continuous and objective activity data and are associated with muscle weakness and functional outcomes [9]. Comparing subjective scores (RATs and MQS) with accelerometry may help establish convergent validity and improve the precision of activity assessments. Moreover, the 2018 PADIS guidelines emphasize early mobilization and optimization of the activity dose, highlighting the need for standardized and reproducible measurement methods [10]. Progressive early mobilization programs have also been linked to clinical and economic benefits [11].

However, few studies have simultaneously evaluated subjective and objective activity measures or examined their association with clinical outcomes. Therefore, this prospective observational pilot study aimed to evaluate the feasibility of recording the Rehabilitation Activity Time score (RATs), the Mobilization Quantification Score (MQS), and wrist-worn accelerometry in mechanically ventilated ICU patients; to examine the convergent validity of RATs against MQS and accelerometer-derived activity data; and to explore associations between activity measures and relevant clinical outcomes.

## Materials and methods

Study design and setting

This prospective, single-center, observational pilot study was conducted in the ICU of a tertiary care hospital - National Hospital Organization Nagoya Medical Center, Nagoya, Japan - between June 2024 and May 2025. The primary objective of this study was to assess the feasibility of daily assessment of rehabilitation dose using both subjective and objective activity measures in mechanically ventilated patients and to explore the convergent validity of these measures. Consecutive eligible patients were enrolled during the study period, and no interventions beyond usual care were introduced; all rehabilitation activities were performed according to routine clinical practice and at the discretion of the treating therapist. This study was conducted and reported in accordance with the Strengthening the Reporting of Observational Studies in Epidemiology (STROBE) guidelines [12].

Participants

Adult patients admitted to the ICU were eligible if they were aged 18 years or older, required, or were anticipated to require invasive mechanical ventilation for at least 48 hours, and were scheduled to receive rehabilitation therapy during their ICU stay as part of their usual care. Eligibility was assessed by the treating intensivist and physical therapist within 24 hours of ICU admission based on expected clinical course and rehabilitation planning. Patients were excluded if they had severe neuromuscular disorders that made the assessment of voluntary movement extremely difficult, limb amputation, or skin conditions that prevented safe placement of the accelerometer, predetermined treatment limitations such as withdrawal of care, or if informed consent could not be obtained from the patient or a legal surrogate. Eligible patients or their proxies were approached for participation, and written informed consent was obtained prior to enrollment.

Activity measurements

Physical activity was assessed using two therapist-recorded subjective scores and one objective accelerometry-based measurement. Daily activity scores were recorded once a day by the treating physical therapist based on all rehabilitation sessions performed that day, including bed-based exercises, sitting, standing, and walking. RATs quantifies the duration of the highest activity level achieved during the day, whereas the MQS combines the intensity and duration of activities into a composite dose score [4, 5, 7]. Both scales evaluate activity based on the mobility level and time to produce a numerical representation of the rehabilitation dose. To minimize variability, all therapists received standardized training using a predefined manual and participated in calibration sessions before data collection; the same scoring method was used throughout the study. All rehabilitation interventions were delivered as part of routine clinical care, without any protocolized modification for study purposes, and the content and intensity were determined solely by the treating therapists. The detailed scoring rubric and operational definitions for the RATs have been previously developed and are provided in the referenced manual [8], which was used consistently throughout the study.

Objective activity was measured using a wrist-worn triaxial accelerometer (ActiGraph wGT3X-BT; Ametis, Pensacola, Florida) on the non-dominant side. The device was worn continuously throughout the ICU stay whenever possible, and any removal or interruption was documented with the corresponding reason. A valid wear day was predefined as ≥20 hours of recorded data, consistent with prior ICU accelerometry studies. For each valid day, vector magnitude (VM), defined as the square root of the sum of the squared activity counts from the three axes, was calculated and used as the primary movement metric. From these VM data, we derived the daily total VM (counts/day) [13]. Accelerometer data were processed using standard ActiGraph procedures without the application of activity intensity cut-points, given the exploratory nature of this pilot study. Raw activity counts were summarized as daily total vector magnitude (counts/day) to represent overall movement volume.

Clinical data and outcomes

Baseline demographic and clinical data were extracted from the medical records, including age, sex, primary diagnosis, the Acute Physiology and Chronic Health Evaluation II (APACHE II) score at ICU admission, and the Sequential Organ Failure Assessment (SOFA) score at extubation.

Clinical outcomes included ICU length of stay, duration of mechanical ventilation, Medical Research Council (MRC) score at ICU discharge [14], and Barthel Index at hospital discharge [15]. The MRC score was assessed at ICU discharge, and the Barthel Index was evaluated at hospital discharge by trained clinicians as part of routine functional assessment.

Statistical analysis

This study was designed as a pilot investigation; therefore, a target sample size of 10 patients was selected to evaluate feasibility and generate preliminary estimates of effect sizes for future large-scale studies. No a priori sample size calculation was conducted. Continuous variables are reported as medians with interquartile ranges (IQRs), while categorical variables are summarized as frequencies and percentages. No imputation was performed for missing data, and analyses were conducted using available-case data only, consistent with the descriptive and feasibility-focused aims of this pilot study.

Feasibility was assessed by examining multiple aspects of the data collection process, including the daily recording rates of the RATs and MQS, duration of accelerometer wear and proportion of valid days, frequency and reasons for missing data, and occurrence of any adverse events or skin complications related to accelerometer use.

To examine the convergent validity of the RATs against established activity measures, correlations among the RATs, MQS, and accelerometer-derived vector magnitudes were analyzed using Spearman's rank correlation coefficient. The primary analytical focus was on the strength and direction of correlations, effect sizes, and 95% confidence intervals (CIs) rather than on p-values, consistent with the exploratory nature of this pilot study.

Although statistical significance was defined as p<0.05, hypothesis testing was not emphasized as the study aimed to describe preliminary trends and assess feasibility rather than to establish causal inferences. All analyses were performed using JMP software, Japanese version (version 13.0; SAS Institute Inc., Cary, North Carolina).

Ethical considerations

This study involved no interventions outside standard clinical care and was conducted in accordance with the ethical principles of the Declaration of Helsinki and relevant national regulations. Ethical approval was granted by the Institutional Review Board of Nagoya Medical Center (approval number: 2023-007). Written informed consent was obtained from all participants or their legally authorized representatives prior to study participation. All patient information was anonymized and used solely for research purposes.

## Results

Patient characteristics

During the study period, 12 patients were screened; among them, 10 were enrolled. Two patients were excluded as they failed to provide informed consent (n=2). The median age of the included patients was 68 years (IQR, 61-81 years), and eight patients (80%) were men. The median APACHE II score at ICU admission was 22 (IQR 18-34), and the median SOFA score at extubation was 11 (IQR 7-13). Primary diagnoses included sepsis (n=4), respiratory failure (n=3), heart failure (n=2), and postoperative management (n=1). The median length of ICU stay was six days (IQR 4-7), and the median duration of invasive mechanical ventilation was four days (IQR 2-5). The median MRC score at ICU discharge was 43 (IQR 35-50), and the median Barthel Index score at hospital discharge was 80 (IQR 72-96). Patient characteristics are summarized in Table 1.

**Table 1:** Patient characteristics IQR - interquartile range; APACHE II - Acute Physiology and Chronic Health Evaluation II; SOFA - Sequential Organ Failure Assessment; MRC - Medical Research Council

Variable	Value (n=10)
Age, years, median (IQR)	68 (61–81)
Male sex, n (%)	8 (80)
APACHE II score, median (IQR)	22 (18–34)
SOFA score at extubation, median (IQR)	11 (7–13)
Primary diagnosis, n (%)	
Sepsis	4 (40)
Respiratory failure	3 (30)
Heart failure	2 (20)
Postoperative management	1 (10)
ICU length of stay, days, median (IQR)	6 (4–7)
Duration of mechanical ventilation, days, median (IQR)	4 (2–5)
MRC score at ICU discharge, median (IQR)	43 (35–50)
Barthel Index at hospital discharge, median (IQR)	80 (72–96)

Feasibility of activity measurements

A total of 59 patient-days of ICU stay were recorded. The RATs were documented on 56 of 59 days (95%), whereas the MQS was recorded on 53 of 59 days (90%). The accelerometer was applied to all 10 patients, and valid recordings (≥ 20 hours/day) were obtained on 55 days (83%). The missing data were mainly due to the absence of therapists on weekends or emergent clinical procedures. No adverse skin events, device malfunctions, or session interruptions were observed. These results indicated that all three measures, that is, RATs, MQS, and accelerometry, were feasible and safe to perform in the ICU environment (Table 2).

**Table 2:** Feasibility of activity measurements in the ICU ICU - intensive care unit; RATs - Rehabilitation Activity Time score; MQS - Mobilization Quantification Score

Item	Value
Total ICU observation days	59 days
RATs recorded, n (%)	56 (95)
MQS recorded, n (%)	53 (90)
Accelerometer worn, n (%)	10 (100)
Valid accelerometer days (≥20 h/day), n (%)	55 (93)
Main reasons for missing data	Therapist absence, emergent procedures
Adverse events related to accelerometer use	0 (0)
Sessions requiring interruption or discontinuation	0 (0)

Correlation among activity measures

Based on Spearman's rank correlation, the RATs and MQS demonstrated a very strong positive correlation (r=0.94, 95% CI 0.90-0.99). The RATs also showed a strong correlation with accelerometer-derived daily activity counts (r=0.76, 95% CI 0.26-0.94). Similarly, the MQS was strongly correlated with accelerometer activity counts (r=0.81, 95% CI 0.36-0.95). These findings support the convergent validity of the RATs with the MQS and objective activity data (Table 3).

**Table 3:** Correlation between activity measures Note: Correlation coefficients were calculated using repeated-measures Spearman's correlation. RATs - Rehabilitation Activity Time score; MQS - Mobilization Quantification Score; CI - confidence interval

Pair of measures	Correlation coefficient (r)	95% CI
RATs vs MQS	0.94	0.90-0.99
RATs vs accelerometer activity counts/day	0.76	0.26-0.94
MQS vs accelerometer activity counts/day	0.81	0.36-0.95

Exploratory analysis of activity and clinical outcomes

Exploratory analyses revealed positive associations between the activity measures and clinical outcomes. The mean RATs moderately correlated with the Barthel Index at discharge (r=0.56) and weakly correlated with the MRC score at ICU discharge (r=0.26). The mean MQS was moderately correlated with both the Barthel Index (r=0.50) and the MRC score (r=0.34). The accelerometer-derived mean activity counts demonstrated strong positive correlations with both the MRC score (r=0.94) and the Barthel Index (r=0.85). Because of the limited sample size, statistical significance testing was not performed, and the results were interpreted based on the correlation strength and effect size. The exploratory associations are presented in Table 4.

**Table 4:** Exploratory association between activity measures and clinical outcomes Note: Owing to the small sample size, formal significance testing was not performed. Interpretation was based on correlation strength and effect size. RATs - Rehabilitation Activity Time score; MQS - Mobilization Quantification Score; ICU - intensive care unit

Activity measure	Clinical outcome	Statistic (r)	Interpretation
Mean RATs	MRC score at ICU discharge	r=0.26	-
	Barthel Index at discharge	r=0.56	Moderate positive correlation
Mean MQS	MRC score at ICU discharge	r=0.34	-
	Barthel Index at discharge	r=0.50	Moderate positive correlation
Mean accelerometer activity	MRC score at ICU discharge	r=0.94	Strong positive correlation
	Barthel Index at discharge	r=0.85	Strong positive correlation

## Discussion

In this prospective pilot study, we examined the feasibility and convergent validity of RATs as a novel bedside tool for quantifying rehabilitation doses in mechanically ventilated ICU patients. RATs and MQS were recorded on more than 90% of the ICU days, and accelerometer data were valid for over 80% of the monitored days, confirming the high feasibility of these activity assessments. RATs demonstrated a very strong correlation with the MQS and a strong correlation with accelerometer-derived activity counts, supporting its convergent validity. Collectively, these findings indicate that RATs is a simple, reliable, and clinically meaningful tool for assessing the rehabilitation activity in critically ill patients.

A very strong correlation between RATs and MQS was expected, as both indices integrate activity intensity and duration. The MQS has previously been shown to be associated with clinical outcomes and is widely used in ICU rehabilitation research [4, 5, 7]. However, the MQS requires detailed documentation and calculations, which can limit its use in daily clinical practice. In contrast, RATs focuses on the duration of the highest mobilization level achieved each day, allowing for rapid bedside recording with minimal burden. Despite its simplified scoring structure, RATs retained strong measurement fidelity, demonstrating that it can serve as a practical alternative to the MQS with greater feasibility for routine implementation.

The strong association between RATs and accelerometer-based measurements further supports its construct validity. Accelerometers provide objective, continuous data and have been linked to muscle strength and functional outcomes [16]. However, their routine use in ICUs is limited by cost, data handling, and technical requirements. RATs can be completed quickly by bedside therapists without specialized equipment or analysis, thereby offering a more accessible approach. Although RATs may not capture subtle intra-day fluctuations, its strong correlation with accelerometer-derived activity suggests that it reflects the overall activity dose with sufficient accuracy for clinical and research use.

This study also provides preliminary evidence suggesting that higher activity levels are associated with more favorable outcomes, including higher Barthel Index scores and earlier ventilator liberation. These trends are consistent with those of previous research demonstrating that early and progressive mobilization contributes to improved physiological recovery and functional independence [1,2,17]. The biological plausibility of these findings is supported by the known effects of physical activity on muscle strength, respiratory mechanics, and neuromuscular function, which facilitate ventilator weaning and recovery.

A major strength of this study is that both subjective (RATs and MQS) and objective (accelerometry) measures of activity were evaluated simultaneously in the same cohort. This is the first study to prospectively assess RATs in mechanically ventilated ICU patients and to demonstrate its feasibility, validity, and potential clinical relevance. However, this study had several limitations. The small sample size and single-center design limit the generalizability, and no repeated-measures or multivariable analyses were conducted because of the pilot nature of the study. Activity scores were recorded once daily and may not reflect intra-day variability. Confounding factors such as illness severity or sedation level were not adjusted for, and wrist-worn accelerometers may have underestimated lower limb movements. Therefore, a causal relationship could not be established. Accordingly, the findings of this pilot study should be interpreted as hypothesis-generating rather than confirmatory.

Despite these limitations, RATs appears to be a promising tool for the standardized quantification of the rehabilitation dose in critically ill patients. Future multicenter studies with larger sample sizes should validate RATs, establish clinically meaningful thresholds, and assess its responsiveness over time. Integration into ICU rehabilitation protocols or electronic medical records may support real-time dose monitoring and individualized rehabilitation strategies. Ultimately, the use of RATs in larger interventional trials could help clarify dose-response relationships and promote evidence-based optimization of ICU rehabilitation practices. 

## Conclusions

The RATs demonstrated high feasibility and strong validity for quantifying rehabilitation doses in mechanically ventilated ICU patients. The RATs was strongly correlated with the MQS and accelerometer-based activity, reflecting overall physical activity levels while remaining simple for bedside use. As a practical and reliable assessment tool, the RATs may facilitate the standardized evaluation of rehabilitation intensity and support future studies exploring dose-response relationships in ICU rehabilitation.
